# The molecular regulation of autophagy in antimicrobial immunity

**DOI:** 10.1093/jmcb/mjac015

**Published:** 2022-03-12

**Authors:** Chuan Qin, Yalan Lu, Lin Bai, Kewei Wang

**Affiliations:** Institute of Laboratory Animal Sciences, Chinese Academy of Medical Sciences & Comparative Medical Center, Peking Union Medical College, Beijing 100021, China; Institute of Laboratory Animal Sciences, Chinese Academy of Medical Sciences & Comparative Medical Center, Peking Union Medical College, Beijing 100021, China; Institute of Laboratory Animal Sciences, Chinese Academy of Medical Sciences & Comparative Medical Center, Peking Union Medical College, Beijing 100021, China; Institute of Laboratory Animal Sciences, Chinese Academy of Medical Sciences & Comparative Medical Center, Peking Union Medical College, Beijing 100021, China

**Keywords:** autophagy, microbe, antimicrobial immunity, endoplasmic reticulum stress, unfolded protein response, apoptosis, infectious disease

## Abstract

Autophagy is a catabolic process that can degrade worn-out organelles and invading pathogens. The activation of autophagy regulates innate and adaptive immunity, playing a key role in the response to microbial invasion. Microbial infection may cause different consequences such as the elimination of invaders through autophagy or xenophagy, host cell death, and symbiotic relationships. Pathogens adapt to the autophagy mechanism and further relieve intracellular stress, which is conducive to host cell survival and microbial growth. The regulation of autophagy forms a complex network through which host immunity is modulated, resulting in a variety of pathophysiological manifestations. Modification of the autophagic pathway is an essential target for the development of antimicrobial drugs.

## Introduction

Microbial pathogens include protozoa, fungi, bacteria, and viruses (Manzanillo et al., 2012; Oikonomou et al., 2016; Maronek et al., 2020). Their invasion alters the local environment in the context of host–pathogen, leading to a series of changes such as reactive oxygen species (ROS) production, endoplasmic reticulum (ER) stress, unfolded protein response (UPR), autophagy, and immunomodulation (Huang et al., 2013a; Liang et al., 2019a). Intracellular stress interferes with protein synthesis, energy metabolism, cell cycle, and cytokinesis. When detrimental insults are accumulated to reach a critical threshold, cell death may occur in different ways such as apoptosis, necroptosis, and necrosis (Liang et al., 2014; Wang et al., 2020). Clinically, microbial infection manifests diverse characteristics due to the comprehensive effect of different mechanisms such as autophagy, antimicrobial immunity, inflammation, and apoptosis.

**Figure 1:**
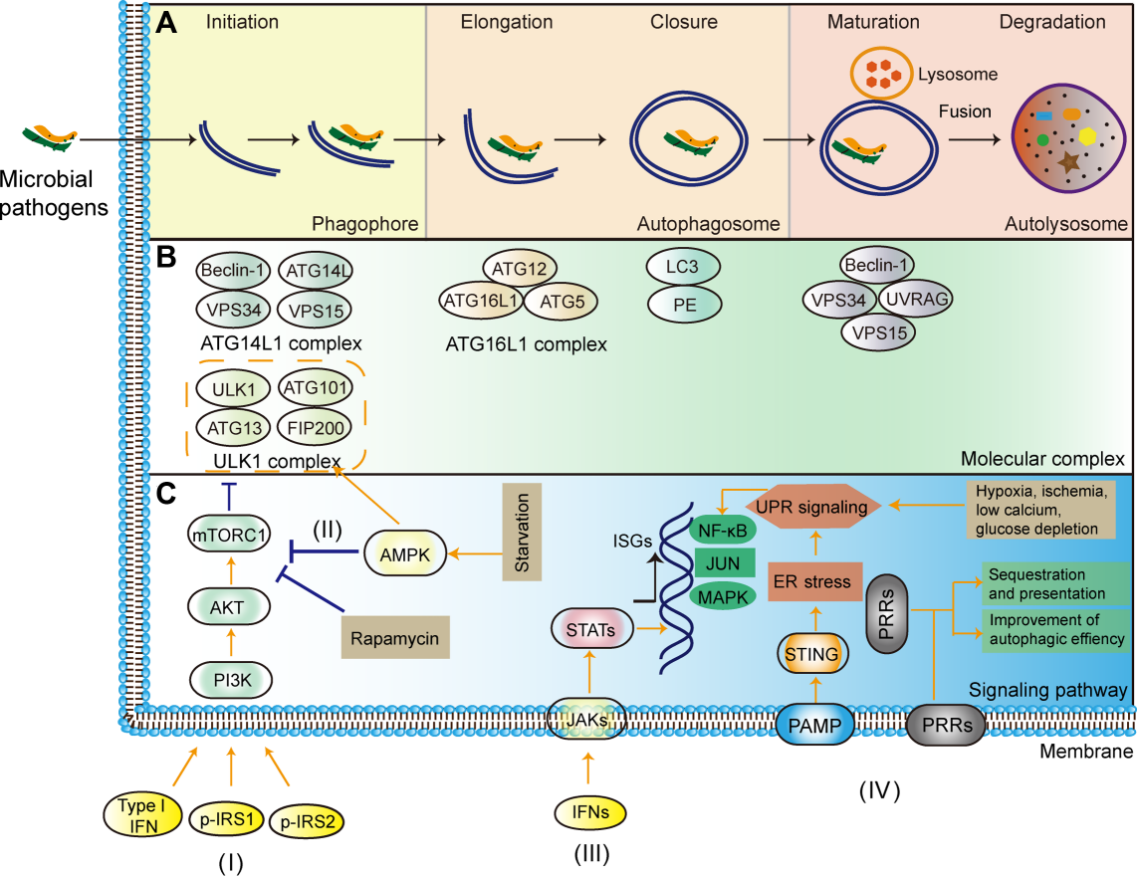
Microbial invasion and activation of autophagy (xenophagy). Autophagy activation consists of several stages such as phagophore formation, fusion with lysosome, and degradation. (**A**) The inaugural phagophore describes a sac-like membrane structure formed in the early stage of autophagy. Phagophore elongation undergoes a sequence of ubiquitination-like reactions. The closed sequestration membrane leads to the formation of a vesicle or autophagosome, which then attaches or fuses with the lysosome. Lysosomal enzymes degrade the content of the autophagosome through a series of hydrolysis reactions. It has been demonstrated that many subcellular compartments, such as the ER–Golgi intermediate compartment (ERGIC), play a role in autophagosome biogenesis during pathogen infection. For example, the ERGIC serves as the membrane source for WIPI2 recruitment and light chain 3 (LC3) lipidation, leading to the formation of autophagosomes that target cytosolic DNA or DNA viruses for degradation by the lysosome. (**B**) ATG proteins and their complexes are involved in different stages. In the initiation of autophagy, the unc-51-like kinase 1 (ULK1) complex (ULK1, ATG101, ATG13, and FIP200) is translocated into the ER, which recruits the ATG14L1 complex (Beclin-1, ATG14L, VPS34, and VPS15). These complexes participate in autophagosome formation and generate ER-associated curly structure to surround invading pathogens. Elongation and closure require the ATG16L1 complex (ATG16L1, ATG12, and ATG5) to specify the site of LC3 lipidation for membrane biogenesis. The autophagosome is fused with the lysosome for further degradation. The Beclin-1/VPS34/VPS15/UVRAG complex involves a hydrolysis process. (**C**) Autophagy-mediated antimicrobial immunity is regulated by different pathways, such as phosphoinositide 3-kinase (PI3K) (I), adenosine monophosphate-activated protein kinase (AMPK) (II), Janus kinase/signal transducer and activator of transcription (JAK/STAT) (III), and ER stress/UPR/pattern recognition receptor (PRR) signaling (IV). The PI3K pathway is activated by type I interferon (IFN), phosphorylated insulin receptor substrate 1 (p-IRS1), and p-IRS2. Its downstream substrate AKT further acts on mammalian target of rapamycin (mTOR) complex 1 (mTORC1). The latter can inhibit autophagy by reducing ULK1 complex recruitment. Rapamycin induces autophagy through the inhibition of mTOR. The activation of AMPK stimulates autophagy by inhibiting mTOR. CD40-mediated autophagy involves the phosphorylation of AMPK at Thr172 and the AMPK-dependent phosphorylation of ULK1 at Ser555. When IFNs bind to membrane receptors, JAKs phosphorylate the tyrosine residue and further STATs are translocated into the nucleus. This process can induce the transcription of IFN-stimulated genes (ISGs) for pathogen killing in the infected cell. ER stress can stimulate UPR signaling. Chronic UPR signaling may involve immunomodulatory crosstalk through pathways such as mitogen-activated protein kinases (MAPKs), JNK, and p38/NF-κB. PRRs recognize antigenic proteins on special epitopes for consequent sequestration and presentation. STING, stimulator of IFN genes; PAMP, pathogen-associated molecular pattern.

Autophagy consists of four basic steps, including initiation, membrane elongation, maturation/fusion, and degradation (Figure 1A; Tanida, 2011). Functional autophagy can be roughly classified into nonselective autophagy and selective autophagy (Periyasamy et al., 2020). Nonselective autophagy is a bulk degradation mechanism, which sequesters a portion of the cytoplasm into autophagosome and mediates global turnover of cytoplasmic components. Selective autophagy recruits specific cargoes that may be tagged with matched ubiquitin, recognized by autophagy adaptor molecules, and then incorporated into autophagosomes for degradation (Sharma et al., 2018; Johansen and Lamark, 2020). Selective autophagy is involved in the regulation of antimicrobial immune processes, including inflammation and cell death. In the autophagy pathway, there are various regulators, such as ATG proteins, Beclin-1/Bcl-2, and ubiquitin-binding adaptors. Accompanied by microbial infection, the activation of autophagy modulates the defense mechanism of host cells, which may increase, decrease, or even skew the immune response. It is worth noting that the induction of autophagy can promote or hinder antimicrobial immunity, showing a dual effect.

## Regulators of functional autophagy

The functional activity of autophagy relies upon autophagy-related proteins and/or their complexes, which are regulated by an intricate network composed of signal constituents and mediators. Several regulators of functional autophagy are summarized as follows.

### ATG proteins

Based on genetic hierarchy, there are multiple ATG proteins to modulate the autophagic process. These proteins generally appear as conjugation or complex to perform functional activities (Mizushima, 2019). In mammals, the ULK1 complex composed of ULK1, FIP200, ATG13, and ATG101 is activated in the initial stage of autophagy, which binds to and phosphorylates ATG9 on the vesicle (Figure 1B). Then, the complex is translocated into the ER to join the ATG14L1 complex composed of ATG14L, Beclin-1, VPS34, and VPS15. After the ATG14L1 complex is recruited to the phagophore assembly site, the ATG12–ATG5–ATG16L1 complex further combines ATG8 (LC3) with phosphatidylethanolamine (PE) (Li and Zhang, 2019). ATG8 and ATG12 are covalently conjugated to PE and ATG5, respectively, which develops an ATG conjugation to take part in the formation of an autophagosome (Mizushima, 2019). ATG conjugation affects antimicrobial immunity by reorganizing intracellular membranes and targeting downstream effectors, which involves canonical and noncanonical autophagy pathways (Choi et al., 2014; Selleck et al., 2015). Of note, the roles of some signal molecules such as ATG5 and ATG8 have been well known in the autophagic process, but ATG proteins have a non-autophagy function as well (Subramani and Malhotra, 2013; Choi et al., 2014; Mizushima, 2019).

### Beclin-1/Bcl-2

Essential Beclin-1 interacts with Bcl-2 to mediate the integration between autophagy and antimicrobial immunity (Casalino-Matsuda et al., 2015). Bcl-2 negatively regulates Beclin-1-dependent autophagy, which determines the fate of host cells (Pattingre and Levine, 2006). Bcl-2 binds to NAF-1 that is interrelated with Beclin-1 in the lumen of the ER. Beclin-1 is dissociated from Bcl-2 to initiate autophagy. Thus, Bcl-2 inhibits Beclin-1-mediated autophagy only in the ER. There is a molecular crosstalk between autophagy and apoptosis, which provides mechanistic insights under ER stress (Maiuri et al., 2010; Wang, 2016). Beclin has a binding domain to interact with the cell-death regulator Bcl-2, which plays an antiviral role in host defense against Sindbis virus infection (Liang et al., 1998). Beclin-1 also binds to the infected cell protein 34.5 (ICP 34.5) of herpes simplex virus type 1 (HSV-1) to mediate innate immunity and fatal infection (Leib et al., 2009).

### RUN domain Beclin-1-interacting cysteine-rich domain-containing protein

RUN domain Beclin-1-interacting cysteine-rich domain-containing (Rubicon) protein is a binding partner of the Beclin-1–VPS34-containing complex negatively to regulate the maturation step of autophagy as well as to mediate the activation of phagocytic nicotinamide adenine dinucleotide phosphate (NADPH) oxidase (Yang et al., 2012a). Upon microbial infection or the activation of toll-like receptors (TLRs), Rubicon interacts with p22*^phox^* of the NADPH oxidase complex to induce the blowout of antimicrobial ROS and inflammatory cytokines. Therefore, Rubicon may be required to embody optimal immunity against microbial infection. Rubicon is also a feedback inhibitor of CARD9-mediated innate immunity (Yang et al., 2012b). Rubicon acts on autophagy complex and phagocytosis complex, which is mainly related to LC3-associated phagocytosis (LAP). Rubicon may thus be pivotal to targeting intracellular signaling complexes and generating an optimal immune response against microbial infections (Yang et al., 2012a, b; Liang et al., 2014).

### Redox regulation

The autophagic process is modulated by nitric oxide, hydrogen peroxide, electrophiles, etc. These regulators form intracellular redox networks via covalently binding to the domains of cysteine-containing and redox-sensing proteins, including the Keap1–Nrf2 pathway and thiol–electrophile interactions (Levonen et al., 2014). Redox regulation mediates the interaction between Nrf2/Keap1 antioxidant and p62/sequestosome 1 (SQSTM1)-associated autophagy. p62 adapts oxidized Keap1 to autophagic degradation, thereby quickly recovering the Nrf2/Keap1 system (Levonen et al., 2014). Besides, the cysteine protease ATG4 is involved in the conversion of LC3I to LC3II. ATG5 and ATG7 are redox-dependent during the shear stress-mediated autophagic activity (Liu et al., 2015). Cysteine-rich autophagy proteins can be modified by reactive species such as –SOH, –SO_2_/3H, –S-lipid, –SSG, and –SNO (Levonen et al., 2014). Redox regulation modulates the autophagy pathway, as revealed in the pathogenesis of certain diseases (Rose and Hoffmann, 2015; Fertan et al., 2019). Redox regulation mediates ROS production, oxidative stress, energy metabolism, and so forth, which may indirectly influence the host innate and adaptive immunity.

### DNA sensors

Upon binding to microbial DNA, cyclic guanosine monophosphate–adenosine monophosphate (cGAMP) synthase (cGAS) produces cGAMP to induce the LC3 lipidation through WIPI2- and ATG5-dependent pathways (Gui et al., 2019). The cytosolic sensor cGAS is enhanced by *Escherichia coli* DNA treatment, which promotes autophagic response through the MyD88-independent TLR4 signaling pathway (Wang et al., 2019a). The cGAS–Beclin-1 interaction increases the autophagic degradation of invader DNA to maintain an appropriate balance during antimicrobial immunity (Liang et al., 2014). The sensor cGAS can bind to *Mycobacterium tuberculosis* DNA in macrophages, which induces IFN production via the STING/TANK-binding kinase 1 (TBK1)/IFN response factor 3 (IRF3) pathway against *M. tuberculosis* (Watson et al., 2015). Other double-strand DNA sensors such as IFI204 and AIM2 also take part in the regulation of autophagy-involved immunity as indicated by IFN-β release or interleukin-1β (IL-1β) production during *Mycobacterium bovis* infection (Chunfa et al., 2017).

### Inhibitor of apoptosis proteins

In cytokinesis, survivin/BIRC5 can bind to ATG5 to interfere with the correct assembly of the chromosome passenger complex (Simon and Friis, 2014). AKT/mTOR/survivin signaling may reverse the autophagy-induced survival mechanism (Pei et al., 2015). X-linked inhibitor of apoptosis protein (XIAP) can be directly targeted by miR-192-3p at the 3′-untranslated region. The miR-192-3p/XIAP axis promotes hepatitis B virus (HBV) replication via autophagy activation (Wang et al., 2019b). Phosphorylated XIAP controls the starvation-caused autophagy downstream of the PI3K/AKT pathway. There is a novel XIAP–Mdm2–p53 pathway to inhibit autophagy (Huang et al., 2013b). The NOD2–RIPK2–XIAP pathway regulates antibacterial autophagy in patients with Crohn's disease (Schwerd et al., 2017). Both XIAP and cIAP1 can activate NF-κB signaling by binding p65 to the promoter of Beclin-1 for transcriptional regulation (Lin et al., 2015).

### Immune factors

Autophagy activation modulates antimicrobial immunity, which is regulated by the feedback of immunological factors (i.e. immunoglobulins and cytokines). Intravenous immunoglobulin increases the formation of autophagosomes to kill multidrug-resistant *E. coli* and *Pseudomonas aeruginosa* in the cytoplasm of neutrophils (Matsuo et al., 2015; Das et al., 2020). Th1 cells produce the cytokine IFN-γ to inhibit bacterial infection via the activation of autophagy (Ohshima et al., 2014). During the infection of *M. tuberculosis* in macrophages, the Th1 cytokine IFN-γ stimulates autophagy, whereas Th2 cytokines IL-4 and IL-13 suppress autophagic activity (Ghadimi et al., 2010). In addition, the antimicrobial LL-37 peptide stimulates autophagy activation in macrophages infected with *M. tuberculosis* (Rekha et al., 2015). The release of IFN-β is associated with immunoevasion adopted by *M. tuberculosis* for its intracellular survival (Sabir et al., 2017).

### NOD-like receptors

NOD-like receptors (NLRs) have diverse functions in microbial sensing and antimicrobial immunity by sharing NBD, LRR, and TLR domains (Elinav et al., 2011). The inhibition of the NLRP3 inflammasome involves IFN-β-mediated immunoevasion during *M. tuberculosis* infection (Sabir et al., 2017). The activation of NLRP3 regulates noncanonical fungal autophagy through the IFN-γ-mediated ATF6–C/EBP-β–DAPK1 axis (Oikonomou et al., 2016). The extracellular leucine-rich domain of TLRs belonging to type I transmembrane proteins triggers the initiation of antimicrobial immunity. TLR homologs or heterodimers recruit different adaptors to the intracellular TIR domain (Everts et al., 2014). Therefore, the TLR family plays a critical role in the recognition and autoregulation of PAMPs and damage-associated molecular patterns. TLR4 ligation is related to p62/LC3 recruitment and canonical autophagosome formation, which contributes to bacterial clearance in the infection of enteropathogenic *E. coli* and *Shigella flexneri* (Lee et al., 2017).

## Autophagy regulates antimicrobial immunity

The interaction between pathogen and host may result in different consequences, such as the elimination of microbial pathogens, host cell death, and symbiosis. Meanwhile, the activation of autophagy is pivotal to the pathogenesis of microbial infection, which involves the regulation of innate and adaptive immunity. Antimicrobial immunity is the resistance to microbial invasion, primarily composed of the intracellular degradation of phagosomes, antigen presentation, and the extracellular secretion of antibiotic peptides.

### Autophagy and innate immunity

During microbial infection, activated autophagy regulates innate immunity as reflected by (i) the recognition of antigen components via PRRs or cargo receptors (Delgado et al., 2009), (ii) the sequestration of special constituents into a sac-like membrane structure and subsequent degradation in autolysosomes (Seay and Dinesh-Kumar, 2007), and (iii) the activation of antimicrobial effectors for the induction of immune response (Delgado et al., 2009).

Cargo receptors perform selective autophagy by sensing cytosolic pathogens or their metabolic products for lysosomal degradation and antigen processing. Common cargo receptors are recapitulated as follows.

*p62/SQSTM1.* The affinity of p62 for ubiquitin is enhanced when Ser403 of the ubiquitin-associated (UBA) motif is phosphorylated (Matsumoto et al., 2011). The oligomeric p62 prefers Lys63-linked chains as well as mono-ubiquitin (Wurzer et al., 2015). The cooperation between the oligomerization of p62 and the dimerization of the UBA domain may obtain the selectivity for ubiquitinated cargos (Wurzer et al., 2015).

*Calcium-binding and coiled-coil domain-containing protein 2 (CALCOCO2)/nuclear dot protein 52 (NDP52).* The coiled-coil region of NDP52 mediates a C-terminal ubiquitin-binding zinc-finger domain (Xie et al., 2015). NDP52 is accumulated on cargos to modify multiple chains, but there is a decreased affinity for Lys48-linked di-ubiquitin chains (Walinda et al., 2014; Xie et al., 2015).

*Tax1-binding protein 1 (TAX1BP1).* TAX1BP1 participates in the sequestration of xenophagy, which can negatively regulate NF-κB by editing the ubiquitination of target molecules (Verstrepen et al., 2011; Nakano et al., 2013). TAX1BP1 interacts with A20 or binds to TRAF6 to suppress NF-κB transcriptional activation, thereby establishing a mechanistic linkage between autophagy-mediated regulation and inflammatory response (Nakano et al., 2013).

*Neighbor of BRCA1 gene 1 (NBR1).* The NBR1 receptor contains LC3- and ubiquitin-binding domains to recruit protein aggregates for autophagic degradation. Also, NBR1 can interact with p62 to form oligomers. Adapter proteins NBR1 and p62 modulate the selective degradation of ubiquitinated targets (Kirkin et al., 2009a, b).

*Optineurin (OPTN).* OPTN recognizes ubiquitinated mitochondria to facilitate their degradation via mitophagy. The phosphorylation of OPTN enhances the efficiency of selective autophagy (Richter et al., 2016). In response to viral RNA, OPTN is translocated into perinuclear vesicles that are positive for ATG9A, which dampens NF-κB and IRF3 signaling pathways through the sequestration of the ubiquitin assembly complex and alters the secretion of downstream proinflammatory cytokines (O'Loughlin et al., 2020). Moreover, OPTN amplifies cargo recognition and prolongs the time interval of cargo degradation (Herhaus and Dikic, 2015).

*Other cargo receptors or sensors.* In mammalian cells, there are diverse receptors or sensors, including BNIP3L or NIX, PEX5, ATG8/LC3/GABARAP, SQST-1, and SEPA-1 (Wu and Cui, 2019; Johansen and Lamark, 2020). BNIP3L is specific for mitophagy (Yao et al., 2021). PEX5 is involved in pexophagy (Zhang et al., 2015). Sometimes, different receptors take part in the joint action of selective autophagy. For instance, ATG8/LC3/GABARAP is able to nucleate the autophagosome to facilitate the formation of autophagosomes (Betin and Lane, 2009).

Particular recognition receptors mediate the autophagic response and further control the intracellular survival of infectious microbes such as *M. tuberculosis, Salmonella, Listeria, Shigella*, human immunodeficiency virus 1 (HIV-1), and Sindbis virus (Ponpuak and Deretic, 2011; Deretic, 2012). The activation of autophagy eliminates microbial pathogens via direct capture and/or the generation of antimicrobial peptides (Ponpuak et al., 2010). The expansion of PRRs is demonstrated by the antimicrobial effect of vitamin D3 on tuberculosis and acquired immunodeficiency syndrome (AIDS) (Deretic, 2012). The multiple types of cargo receptors determine the diversity of selective autophagy, which is associated with the initiation of antimicrobial reaction, the elimination of microbial pathogens, and the balance of the immune response.

During cargo-mediated selective autophagy against microbial invasion, E3 ubiquitin ligase catalyzes ubiquitin transfer to the target substrate, which is called ubiquitination (or ubiquitylation). Ubiquitinated components are then recognized by adapter molecules to facilitate their subsequent delivery to the lysosomes for degradation. The features of E3 ligases would be easily explained using the ubiquitin ligases Parkin and Smurf-1 as examples (Li et al., 2019; Bu et al., 2020). The ubiquitination step of E3 ligases is related to the structural features of adaptor molecules, which are connected to Lys29 or Lys48 with four or more ubiquitin molecules (Ponder and Bogyo, 2007).

Antimicrobial proteins are produced through autophagy-mediated machinery. Bactericidal cryptides are made from cytosolic proteins via the autophagic adaptor p62/SQSTM1 against *M. tuberculosis* (Ponpuak et al., 2010; Ponpuak and Deretic, 2011). Virus-infected cells can produce IFNs, which further activate natural killer cells and macrophages to enhance host defense by upregulating the expression of major histocompatibility complex (MHC) antigens. When STING/TBK1/LC3-associated autophagy in macrophages is inhibited during *M. bovis* infection, the production of IFN-β is also suppressed (Chunfa et al., 2017). IFN-β is linked with the immune evasion adopted by *M. bovis*, which counteracts the effects of IL-1β and IL-18 by increasing the production of IL-10. Moreover, autophagy can affect overlapping signal molecules to mediate innate immunity, including HMGB1, IL-1β, TLRs, and NLRs. Autophagy as an innate immunity paradigm is extending its functional scope through the repertoire of PRRs, E3 ubiquitin ligases, and the secretion of antimicrobial proteins (Deretic, 2012).

### Xenophagy

Xenophagy is the essential process toward eliminating intracellular pathogens through autophagy machinery. Cellular xenophagy is an innate component of the immune response that degrades microbial invaders through cargo receptors/adaptors, such as p62/SQSTM1, CALCOCO2/NDP52, TAX1BP1, NBR1, and OPTN (Birgisdottir et al., 2013; Periyasamy et al., 2020). In general, the xenophagy process is considered to share similar signaling pathways to autophagy (Sharma et al., 2018). Cargo receptors can sense cytosolic pathogens or their metabolic products for subsequent ubiquitination. For example, TAX1BP1 acts, along with the ubiquitin-editing enzyme A20, negatively to regulate NF-κB and IRF3 signaling in innate antiviral immunity (Gao et al., 2011; Verstrepen et al., 2011). The residual recruitment of TAX1BP1 to *M. tuberculosis* can be mediated by ubiquitin-binding zinc fingers, an LC3-interacting region (LIR), or endogenous domain oligomerization (Bell et al., 2021). The oligomerization of receptor p62/SQSTM1 stabilizes its interaction with LC3B and linear ubiquitin (Wurzer et al., 2015). p62 interacts with viral protein VP2 through the ubiquitination at Lys411 for selective autophagic degradation, thereby inhibiting the replication of Avibirnavirus (Li et al., 2020). The NBR1 receptor contains LC3- and ubiquitin-binding domains, which can interact with p62 to form oligomers. NBR1 and p62 recruit protein aggregates for the selective degradation of ubiquitinated targets (Kirkin et al., 2009a). In addition to NBR1, other receptors, including p62, OPTN, and NDP52, also possess the binding motif for LC3 or ubiquitin. Furthermore, p62 and NDP52 participate in mitophagy and other types of selective autophagy. Collectively, the ubiquitination as the ‘eat-oneself’ signal is a critical mechanism in cargo receptor-mediated selective autophagy. In turn, not all selective autophagy pathways require ubiquitination (Padman et al., 2019). Moreover, deubiquitination is implicated in selective autophagy as well (Magraoui et al., 2015). The exact mechanism of cargo receptor-mediated innate immunity remains under investigation. The surface protein Rv1468c of *M. tuberculosis* conducts direct binding of ubiquitin to p62 and takes part in the delivery of mycobacteria into LC3-associated autophagosomes (Chai et al., 2019). Rv1468c/TAX1BP1 is involved in the interaction between autophagy degradation and antigen presentation in infected macrophages (Bell et al., 2021). Further, cargo receptors mediate the secretion of cytokines, including the production of antimicrobial cryptides via p62/SQSTM1 against *M. tuberculosis* and the secretion of IFNs through TRIM21/IRF3 for antiviral action (Ponpuak and Deretic, 2011; Kimura et al., 2015). The capacity of autophagy-related endosomes and lysosomes to generate MHC class II–peptide complexes can coordinate antigen acquisition, the formation of T-cell receptor (TCR) ligands, and the subsequent immune response (Inaba et al., 2000). As mentioned earlier, xenophagy/autophagy is regulated by the functional activities of cargo receptors. These receptors can conjugate to ubiquitous LC3 protein for the upcoming formation of autolysosomes. Meanwhile, the LIR motif ensures the targeting of cargo receptors to LC3 anchored in the phagophore membrane. Comprehensively, LC3-mediated antibacterial effects are mainly modulated by the LAP machinery, although it is also related to canonical autophagy. LC3 interacts with p62 to promote the autophagic degradation of ubiquitinated ‘cargoes’. The LIR motif of p62 forms an intermolecular parallel β-sheet with the β2 strand of LC3B, which reveals a core consensus W-x-x-L motif (Birgisdottir et al., 2013). NBR1 can be recognized by autophagy effectors through the canonical W-x-x-L sequence. The presence of a tryptophan residue in the LIR motif enhances the binding affinity. Different LIRs can interact with LC3B and show unique binding properties (Rozenknop et al., 2011). In addition, NDP52 contains the tripeptide Leu–Val–Val as an atypical LIR motif, which can specifically bind to LC3C for efficient recruitment of ATG8 members to bacteria-degrading autophagosomes (von Muhlinen et al., 2012). TAX1BP1 homologous to NDP52 includes the same atypical LIR motif as NDP52 to bind to ubiquitin (Newman et al., 2012). The additional recruitment of OPTN and NDP52 can amplify mitophagy through an ATG8-dependent positive feedback loop (Padman et al., 2019).

### Autophagy and adaptive immunity

**Figure 2:**
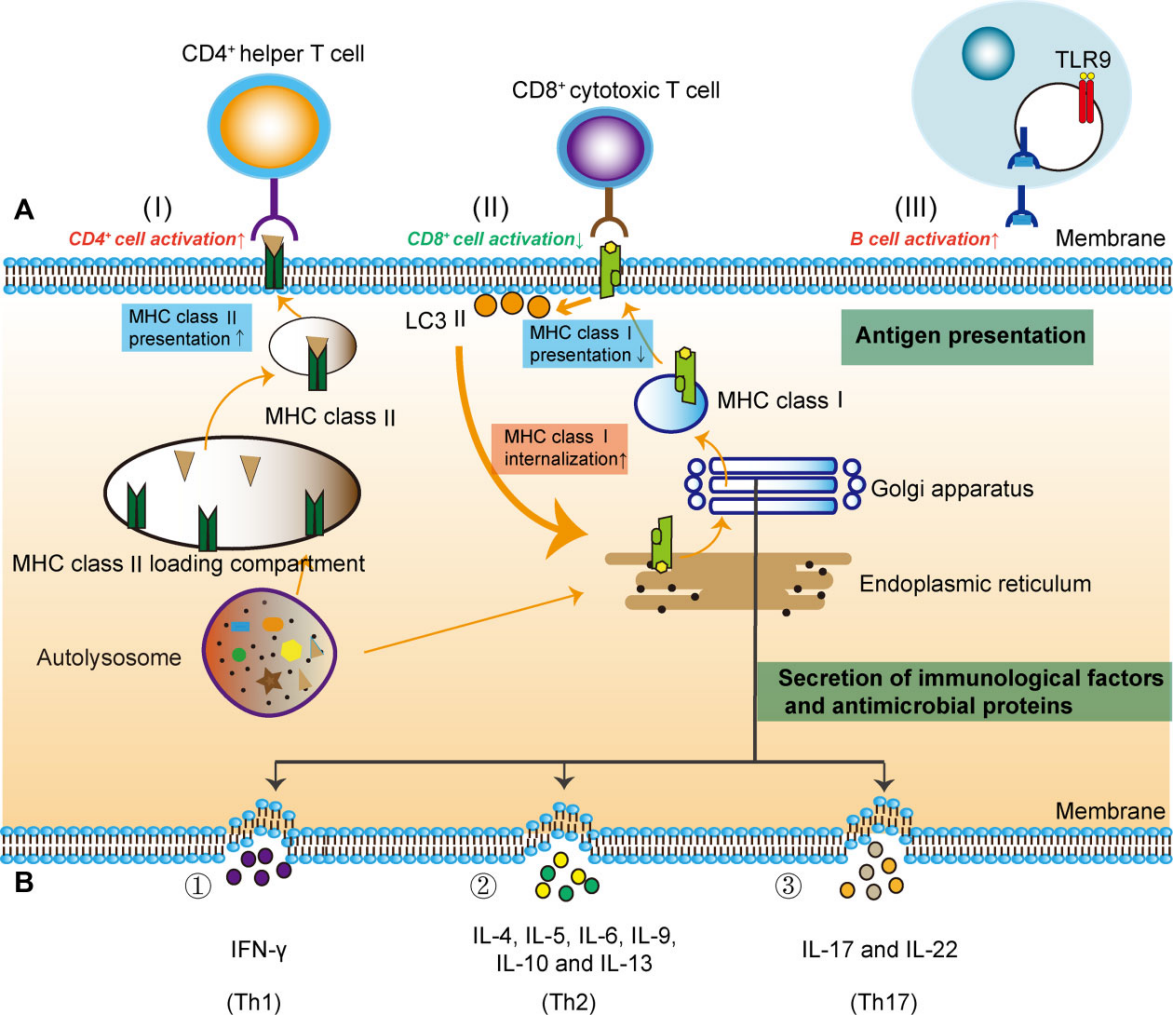
The interaction between autophagy and antimicrobial immunity. Autophagy induces different effects on antimicrobial immunity. For instance, the autophagy activation of monocytes is increased by Th1 cytokine IFN-γ, whereas it is decreased by immunosuppressive Th2 cytokines IL-4 and IL-13. PRRs recognize antigenic peptides for MHC presentation. (**A**) Antigen presentation. Autophagy facilitates MHC class II molecules on antigen-presenting cell surface and stimulates CD4^+^ helper T cells (I). The presentation of MHC class I molecules on an antigen-presenting cell is reduced by increasing internalization of LC3 II molecules (II). In a recent study, it was reported that MHC class I is also a selective substrate of autophagy in tumor immune evasion. Aberrant degradation of MHC class I may be a way for some diseased cells to escape immune recognition. Antigen can be presented to the B-cell receptor by helper T cells, which stimulates TLR9 to activate B cell and humoral immunity (III). (**B**) The secretion of immunity-related proteins. Many cytokines are synthesized in the ER, processed within the ER–Golgi apparatus, and secreted for the regulation of autophagy. Th1 cells produce IFN-γ to inhibit bacterial infection via the autophagic mechanism (①); Th2 cells secrete IL-4, IL-5, IL-6, IL-9, IL-10, and IL-13 to mediate inflammation (②); Th17 cells generate IL-17 and IL-22 to regulate the production of antimicrobial factors (③).

Microbial pathogens are phagocytosed and degraded via the autophagy recycling. Acquired antigens are loaded into MHC-II molecules (Figure 2A). An MHC-II molecule with antigenic peptide is transferred onto the surface by which the antigen is presented to a helper T cell. Activated helper T cells release various cytokines to combat extracellular pathogens. Thereupon, the autophagy machinery functions as an antimicrobial regulator. Autophagy signaling controls the development of adaptive immunity as follows.

*The selection of T cell repertoires in the thymus.* The presentation of endogenous antigens is regulated by the autophagy pathway, which determines the selective survival of some CD4^+^ T cells in the thymus. Organ transplantation has demonstrated that mice bearing an ATG5-deficient thymus develop severe autoimmune disease (Pua et al., 2007). Autophagy conserves the MHC class II–peptide repertoire of thymic epithelial cells (TECs). Self-antigenic epitopes are presented by MHC class II molecules on TECs, which generate a functional and self-tolerant CD4^+^ T cell lineage. Autophagy is essential for endogenous MHC class II loading and T cell selection (Nedjic et al., 2008). The autophagy machinery can skew the immune response as well. Th1 or Th2 bias plays a vital role in the context of immune-related diseases (Shakya et al., 2018).

*The homeostasis of mature T cells.* Autophagy induction in T lymphocytes promotes T cell clonal expansion following antigen stimulation, which is suppressed by the negative feedback loop involving FAS-associated death domain (FADD) and caspase-8. Otherwise, hyperactive autophagy during T cell mitogenesis leads to programmed necrotic death (Walsh and Bell, 2010). Beclin-1 deficiency impairs T cell differentiation and reduces Th1 and Th17 cells (Liu et al., 2013). The autophagy gene ATG5 is necessary for T lymphocyte survival and proliferation (Pua et al., 2007). In ATG5^−^^/^^−^ chimeric mice, the cell death of CD8^+^ T lymphocytes is significantly exacerbated. Moreover, CD4^+^ and CD8^+^ T cells fail to proliferate effectively subsequent to TCR stimulation. ATG7- and ATG3-deficient T cells display phenotypes similar to that of ATG5-deficient T lymphocytes, characterized by increased cell death and the defective homeostasis of organelles such as mitochondria and ER (Jia and He, 2011). The deletion of gene ATG16L1 in mouse macrophages alters the production of IL-1β, which affects the derivation of naïve T cells into Th17 (Kaser and Blumberg, 2011).

*Antigen presentation.* Antigenic peptides generated from autophagic degradation can be presented by MHC class II molecules. CD4^+^ T cells classically recognize these surface antigens that are degraded and processed in lysosomes. In Epstein–Barr virus (EBV) infection, CD4^+^ T cell recognition is sensitized by EBV nuclear Ag (EBNA) epitopes. The suppression of autophagy decreases the recognition by EBNA1-specific CD4^+^ T cell clones (Taylor et al., 2006). In addition, autophagy-mediated antigens modulate adaptive immunity by altering the net outcome for CD4^+^ helper and CD8^+^ cytotoxic T cell responses (Figure 2A; Munz, 2016). *M. tuberculosis* can escape the immune defense of host cells. The mechanism is that the expression of PE_PGRS47 protein inhibits the autophagy of infected phagocytes and diminishes the presentation of MHC class II-restricted antigens (Saini et al., 2016). In HIV-1 target cells, both canonical autophagy and noncanonical autophagy are involved in the presentation of virus-derived antigens. HIV-1 specifically encodes Tat, Nef, and Vpu proteins, which perturb the autophagy mechanism and evade the antiviral responses (Leymarie et al., 2017).

*The development of B cells.* The maintenance of the B-1a B cell number needs the adequate expression of ATG5 during efficient B cell development. ATG5^−^^/^^−^ progenitors show a substantial defect in the transition from pro-B to pre-B cells. While ATG5 is deleted in B lymphocytes, there is a dramatic decline in the number of B-1 B cells (Miller et al., 2008). ATG5^−^^/^^−^ differentiating plasma cells have high expression of the transcriptional repressor Blimp-1 and enhanced immunoglobulin synthesis, which results in autophagy-dependent cell death in mutant plasma cells. A knockout mouse with conditional deficiency of ATG5 in B cells exhibits a small number of long-lived plasma cells and defective antibody response (Pengo et al., 2013). In addition, the survival of memory B cells and their differentiation into plasma cells are affected by autophagic defect in the autophagosome–lysosome fusion as evidenced in Vici syndrome (Piano Mortari et al., 2018).

*The efficiency of vaccines.* Some conjugate vaccines have a high antigen-presenting ability to T cells when processed by autophagy. The enhancement of autophagy has been used as an optimized strategy for the development of new vaccines against Japanese encephalitis virus (Zhao et al., 2019). When the mycobacterial antigen Ag85B was overexpressed in bacillus Calmette–Guérin vaccine, the vaccine efficacy was improved by augmenting autophagy-mediated antigen presentation (Jagannath et al., 2009). The SIVgag–LC3b fusion antigen could be processed by autophagy-mediated degradation and further presented to the MHC-II compartment, which elicited a strong antigen-specific CD4^+^ T cell response and provided an alternative strategy for the development of an effective HIV vaccine (Jin et al., 2014).

## Signaling pathways for autophagy to regulate antimicrobial immunity

**Figure 3:**
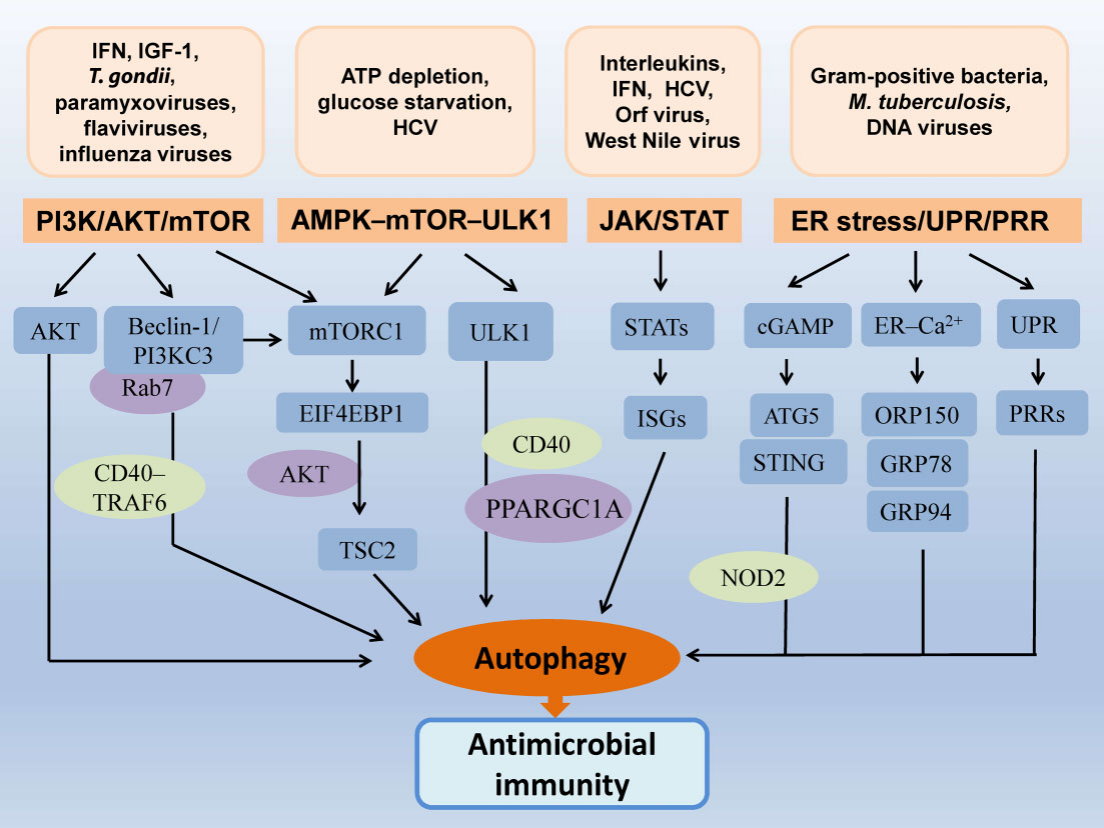
Signaling pathways mediate the interaction between autophagy and antimicrobial immunity. Microbial invasion triggers the activation of autophagy via different signaling pathways by which multiple cargo receptors or sensors are involved through direct or indirect ways. Cytosolic constituents are delivered onto PRRs via topological inversion, acting as the antimicrobial effectors of TLRs. For example, viral nucleic acids can be recognized by intracellular sensors to play a crucial role in the initiation of antigen-specific adaptive immunity. Herein, initiation factors and cargo receptors or sensors are integrated into different signaling pathways to modify antimicrobial immunity. EIF4EBP1, eukaryotic translation initiation factor 4E binding protein 1; GRP78, glucose-regulated protein 78; GRP94, glucose-regulated protein 94; ORP150, oxygen-regulated protein; TSC2, tuberous sclerosis complex 2.

Autophagy regulates antimicrobial immunity through multiple pathways and involves different mechanisms such as genetic traits, inflammation, oxidation, apoptosis, and energy metabolism. For example, microbial invasion can cause the release of inflammatory cytokines and ROS generation (Tse et al., 2004; Levonen et al., 2014; Figure 2B). TNF-α, IL-1β, NO_2_^−^, and O_2_^−^ participate in the coupling of innate and adaptive immunity (Tse et al., 2004). Programmed cell death or apoptosis eliminates intracellular pathogens, which is regulated by IAPs, Bcl-2, caspases, nuclear factors, and so forth (Liang et al., 1998; Lin et al., 2015). There is a complicated network to regulate autophagy-mediated antimicrobial activity, which is validated by representative pathways (Figure 3).

### PI3K/AKT/mTOR pathway

PI3K phosphorylates downstream signal transducers to mediate cell proliferation, differentiation, and intracellular trafficking. The activation of the Beclin-1/PI3KC3 complex recruits ATG proteins onto the isolation membrane in response to stress signals such as starvation, cytokines, and pathogens (Li and Zhang, 2019). The combination of the Beclin-1/PI3KC3 complex and Rab7 can control the fusion of the autophagosome/parasitophorous vacuole with lysosome for the intracellular killing of *Toxoplasma gondii* (El-Awady et al., 2015). The PI3K pathway is activated by innate type I IFN to promote autophagy, which can convert LC3-I to LC3-II for the formation of autophagosomes (Schmeisser et al., 2014). AKT is a downstream substrate of PI3K. PI3K/AKT activation stimulates viral replication and delays virus-induced apoptosis by modulating the autophagy machinery (Schmeisser et al., 2014). PI3K is also activated through p-IRS1 and p-IRS2, which results in the subsequent induction of mTORC1 (Zoncu et al., 2011). The PI3K/mTOR/autophagy pathway participates in host defense against microbial infection. The inhibition of mTOR by rapamycin induces autophagy and incurs immunosuppression (Fabri et al., 2011). mTOR can repress autophagy by phosphorylating ATG proteins. Besides, the PI3K/AKT/mTORC1 pathway is required for the transcription and/or mRNA translation of ISGs, which modulates autophagy and plays an important role in virus clearance and antigen presentation (Schmeisser et al., 2014; Figure 1C). PI3K/AKT/mTOR signaling regulates B lymphocyte proliferation and humoral immunity, as well as T cell activation and cytokine balance (Ma et al., 2019).

### AMPK–mTOR–ULK1 axis

AMPK is a key energy sensor to integrate intracellular energy metabolism. AMPK signaling is activated by ATP depletion or glucose starvation, which can induce autophagy through the inhibition of mTORC1 and direct phosphorylation of the protein kinase Unc-51 ULK1 (Kim et al., 2011). Both the phosphorylation of AMPK at Thr172 and the AMPK-dependent phosphorylation of ULK1 at Ser555 can mediate CD40-stimulated autophagy and intracellular killing of *T. gondii* in the infected cells (Liu et al., 2016). AMPK-activated autophagy contributes to antibacterial defense against *M. tuberculosis* by inhibiting the phosphorylation of mTOR in macrophages. AMPK-mediated antimicrobial activity requires the participation of peroxisome proliferator-activated receptor-γ, coactivator 1α (PPARGC1A) that involves the fusion of phagosomes with LC3B autophagosomes (Yang et al., 2014). In macrophages infected with *M. tuberculosis*, AMPK and PtdIns3K pathways take part in the activation of autophagy induced by antimicrobial LL-37 peptide (Rekha et al., 2015). During hepatitis C virus (HCV) infection, the net strength of autophagy depends on the inhibitive effect of AKT on AMPKα. HCV-induced ER stress impedes the AKT–TSC–mTORC1 pathway, contributing to autophagy enhancement (Huang et al., 2013a). Mechanistic investigation has demonstrated that the activation of AMPK as a new mechanism is influenced by TDRD7 (Subramanian et al., 2018).

### JAK/STAT pathway

JAK/STAT signaling joins autophagy-mediated antimicrobial immunity against microbial pathogens such as West Nile virus, HCV, *Mycobacteria, Shigella*, and *Listeria* (Figure 1C; Ambrose and Mackenzie, 2011; Holla et al., 2014). The inhibition of JAK/STAT signaling decreases sensitivity to internal modulators and contributes to the evasion of autophagy (Holla et al., 2014). A signaling cascade is initiated after external cytokines such as IFNs and ILs as ligands bind to membranous receptors. Receptor-associated JAKs phosphorylate tyrosine residues and then activate STATs to induce the transcription of target genes (Jatiani et al., 2010). There is a cross-regulation between autophagy and type I IFN signaling during host defense. The JAK/STAT pathway is modulated by IFN-induced autophagy, leading to the upregulation of numerous ISGs and virus killing within infected cells. In contrast, many viruses have evolved extraordinary strategies to counteract host immunity by targeting the JAK/STAT pathway (Fleming, 2016). HCV structural proteins block the JAK/STAT pathway, by which IFN-α-mediated STAT activation and the antiviral effect are suppressed. HCV infection can disturb IFN-α signaling and facilitates the escape of HCV from the IFN system, resulting in the persistence of virus infection (Luquin et al., 2007). A similar phenomenon is also found in West Nile virus infection. The viral proteins NS4A and NS4B display a high correlation by inhibiting JAK/STAT signal transduction to IFN-α (Ambrose and Mackenzie, 2011). The JAK/STAT pathway participates in the replication of Orf virus in keratinocytes, which can cause acute pustular skin lesions. Potential mechanisms involve the inhibition of ISGs (e.g. GBP1 and MxA) and the dephosphorylation of STAT1 (Harvey et al., 2015). The JAK/STAT signaling regulates the positive feedback loop for the production of type I IFN, which mediates autophagy induction and virus clearance (Schmeisser et al., 2014). JAK2/STAT1 activation involves HSV-1-induced cellular inflammation that is related to the interaction between IFN-γ and the autophagy mechanism (Chang et al., 2010). Also, JAK/STAT signaling is altered in response to mycobacterial infection that is reported in patients with IFN-γR1 deficiency (Korol et al., 2020).

### ER stress/UPR/PRR signaling

ER stress/UPR/PRR signaling is a network involved in the regulation of autophagy. The vita-PAMP of Gram-positive bacteria mediates ER stress via the innate sensor STING, which triggers autophagy activation to engage immune defense and the homeostatic mechanism (Figure 1C; Moretti et al., 2017). If sustained, the UPR can exacerbate inflammation and it has been implicated in pathologies such as obesity, type 1 and type 2 diabetes, cancer, as well as neurodegenerative and autoimmune diseases. In some cases, ER stress is relieved by microbial infection as revealed in the HCV-infected liver (Dash et al., 2016). HCV infection stimulates autophagic activity and improves the survival of host cells by inhibiting apoptosis. ER stress-induced cellular apoptosis is also ameliorated by activating autophagy in the macrophages infected by *M. tuberculosis* (Liang et al., 2019b; Sathkumara et al., 2021). ER stress-associated UPR signaling can be elicited by different stimuli such as hypoxia, ischemia, low calcium, and glucose depletion (Malhi and Kaufman, 2011; Wang and Kaufman, 2014; Dash et al., 2016). The extent and type of UPR signaling usually depend on pathogen species during the infection (He, 2006). Acute UPR signaling is characterized by returning to baseline level within a short period. Chronic UPR signaling often involves immunomodulatory crosstalk, which may cause an inflammatory response through related pathways such as MAPKs, JNK, and p38/NF-κB (Malhi and Kaufman, 2011; Wang and Kaufman, 2014). Chronic UPR signaling can maintain a persistent state by regulating the expression levels of some genes. Meanwhile, selective autophagy mediated by PRRs plays a crucial role (Fan et al., 2020). PRRs recognize antigenic proteins on special epitopes for consequent sequestration and presentation. During the maturation of T cells, the autophagy machinery loads MHC class I:peptide through ER stress/UPR/PRR signaling, which does not activate cytotoxic T cell response. However, the extracellular part of an MHC class II molecule (i.e. microbial antigens) can be targeted by CD4^+^ T cells, which results in the pathogen being killed. PRR-mediated degradation improves the efficiency of autophagy, which is the most economical way of the development of adaptive immunity (Lin et al., 2018). There is a sequential interplay among ER stress/UPR/PRR signaling, which actually is a compensatory mechanism under stressful conditions. If compensation fails, ER stress-induced apoptosis may be instigated by activating caspase-12 (Szegezdi et al., 2006).

## The paradoxical role of autophagy in antimicrobial immunity

Microbial pathogens induce ER stress and UPR signaling, which may interact with innate sensors to activate selective autophagy and further regulate the immune defense. Therefore, the intracellular fate of invading pathogens is determined by the autophagy-mediated antimicrobial immunity (Ponpuak and Deretic, 2011; Deretic, 2012). The autophagy-dependent eradication of intracellular *T. gondii* is induced by CD4^+^ cell-mediated immunity via CD40/CD154 binding, which is accomplished through the vacuole–lysosome fusion within macrophages (Subauste et al., 2007). Microbial invasion activates autophagy to sequestrate adherent-invasive *E. coli* via the interaction of ATG16L1 with the complement component C3, resulting in the degradation of pathogens (Viret et al., 2020). In response to microbial invasion, vital autophagy can shape the host's immunity to mediate the killing of intracellular pathogens. Instead, certain microbial pathogens may adapt autophagy machinery to favor their survival and growth. At this moment, autophagy becomes a protective mechanism for invading microbes. In host cells infected with *M. tuberculosis*, autophagic adapter protein p62 delivers cytosolic components into autophagosomes (Ponpuak et al., 2010). *M. tuberculosis* can block the fusion of phagosome with lysosomes to keep pathogen survival within conventional phagolysosomes (Vergne et al., 2004). The virulent strains of tuberculosis can boost the production of archetypal Th2 cytokines such as IL-4 and IL-13 to inhibit autophagy in monocytes/macrophages (Harris et al., 2009). Conversely, the autophagy can be stimulated by reducing cytokines IL-4 and IL-13 in response to *M. tuberculosis* antigens (Ghadimi et al., 2010). Also, IFN-β-associated immunoevasion is adopted by *M. tuberculosis*, which protects intracellular bacilli. Evidently, autophagy activity regulated by different cytokines affects the survival of pathogens within host cells (Sabir et al., 2017). Certain bacteria such as *S. flexneri* and *Salmonella typhimurium* can escape antibacterial autophagy and immunity by decreasing the level of cytoplasmic C3-ATG16L1 (Sorbara et al., 2018). Invasive *Shigella* secretes IcsB that competitively inhibits the binding of VirG to ATG5, and thus can avoid autophagic degradation (Ogawa et al., 2005). Intracellular *Listeria monocytogenes* produces pore-forming toxins to impede the maturation of autophagic vacuoles, through which the bacterial pathogen can replicate in vacuoles and establish persistent infection within host macrophages (Birmingham et al., 2008). Autophagy activation may favor the replication of certain viruses. In HCV-infected hepatocytes, autophagy activity is linked to virus replication as reflected by the expression of related proteins such as Beclin-1, ATG4B, ATG5, ATG7, and ATG12 (Ke and Chen, 2014). Autophagy activation alleviates ER stress and regulates the assembly of infectious virions in host cells (Ke and Chen, 2014). Autophagy is a vital mechanism responsible for immunomodulation and immunoevasion (Dash et al., 2016). Moreover, autophagy machinery promotes hepatocyte growth by enhancing phospho-mTOR and 4EBP1 expression. There is a fine balance between HCV invasion and host cell survival, in which autophagy facilitates HCV replication in hepatocytes and develops infectious persistence and pathogenicity (Aizawa et al., 2015). Additionally, HSV-1 protein ICP34.5 binds to Beclin-1 to inhibit the autophagic response. Autophagy inhibition enables viruses to evade innate immunity and causes lethal encephalitis (Orvedahl et al., 2007). Dengue virus can usurp autophagy machinery to acquire free fatty acids from lipid droplets, by which a high level of adenosine triphosphate is provided for virus replication (Heaton and Randall, 2010). Autophagy exerts its unique role to regulate immune homeostasis, which may alter cellular susceptibility to certain pathogens. Intracellular pathogens can exploit host cells and escape antimicrobial degradation, leading to persistent infection and cell death (El-Awady et al., 2015). Obviously, the activation of autophagy acts as double-edged sword, contributing to not only the elimination of pathogens but also immunoevasion and pathogen survival.

Overall, microbial invasion triggers ER stress/UPR signaling and activates autophagy/xenophagy. The perplexing interaction between microbial invasion and autophagic adaptation governs the immunoregulation and controls the final outcome of the host–microbe encounter (Deretic and Levine, 2009). During microbial infection, the host cell adjusts its biomass and function by regulating the autophagy machinery, which involves energy supplementation, modification of innate and adaptive immunity, proliferation, apoptosis, etc. Intracellular pathogens may be eradicated via the autophagic defense mechanism. Alternatively, autophagy machinery can be adapted or subverted to cause persistent infection. By altering or evading autophagy, intracellular invaders can cause host cell death. The pathophysiological manifestations depend on the comprehensive regulation between autophagy and antimicrobial immunity (Yordy et al., 2013).

## Challenge and perspective

### Dual effect and coinfection

The duality of autophagy means that it may have beneficial and harmful effects on the process of antimicrobial immunity. The dual effect of autophagy depends on the trait or nature of microbial pathogens, which has been characterized in the pathogenesis of certain chronic infections such as hepatitis, AIDS, and tuberculosis. When those diseases are treated using autophagy-related medicines, the option of treatment protocols should consider different stages or conditions. For instance, HBV or HBV X protein can activate autophagy in the initial stage, but inhibits subsequent degradation by impairing lysosomal acidification (Khabir et al., 2020). When the autophagy strategy is utilized to treat HBV infection, the pros and cons of autophagy must be weighed. During the coinfection of HBV and hepatitis delta virus (HDV), autophagy proteins such as ATG7, ATG5, and LC3 are implicated in the mutuality of HBV/HDV (Khabir et al., 2020). HDV can alter the autophagic process in the elongation stage to promote genome replication. Therefore, the application of autophagy-related drugs should be cautious in the treatment of HBV/HDV coinfection. Also, the pathophysiological characteristics of clinical disease should be considered. For example, the stable knockdown of several autophagy factors can achieve synergistic inhibition on HIV-1 replication (Eekels et al., 2012). Autophagy thus is a therapeutic target against HIV-1 infection. However, HIV-mediated autophagy/xenophagy flux facilitates the intracellular survival of *M. tuberculosis* or opportunistic pathogens. It is necessary to explore novel strategies under the coinfection of HIV-1 and *M. tuberculosis*. A few drugs such as vitamin D3, trehalose, and phenylbutyrate may be helpful (Rekha et al., 2015; Afsal and Selvaraj, 2016; Sharma et al., 2021).

### Cell death

Essential autophagy modulates both cell survival and death as evidenced through mitochondrial mediation. Mitophagy is a selective degradation of old or damaged mitochondria to maintain survival state. The mitochondrion-dependent pathway is also a well-known mechanism for apoptotic cell death (Wang, 2014). There is a negative feedback loop including FADD and caspase-8, which modulates autophagic response (Walsh and Bell, 2010). Anti-apoptotic Bcl-2 negatively regulates Beclin-1-mediated autophagy and modifies antimicrobial immunity (Casalino-Matsuda et al., 2015). Certain signal molecules are shared by apoptotic and autophagic pathways, including p53, ATG5, caspase-8, Beclin-1/Bcl-2, and IAPs (Miller et al., 2008; Jia and He, 2011; Liu et al., 2013; Pengo et al., 2013; Levonen et al., 2014; Piano Mortari et al., 2018). Autophagy takes part in the degradation of pathogens and subsequent antigen presentation. Apoptosis signaling can provide feedback regulation for autophagic response. The interaction between apoptosis and autophagy is a critical modulator for the functional state of host cells. Excessive apoptosis caused by microbial infection can impair organ function. Therefore, autophagy affects functional maintenance of the infected organ. Autophagic response can activate or antagonize apoptosis, obviously, playing a dual role (Wang, 2015). Necroptosis and pyroptosis are implicated in cell death as well. The enhanced macroautophagic/autophagic activity may be accompanied by necroptosis. The immunogenicity of necroptotic death is a novel strategy for developing cancer vaccines (Lin et al., 2018). The autophagy machinery participates in cytokine storm that destroys a lot of cells in a short time during chimeric antigen receptor T-cell therapy or COVID-19 infection (DeFrancesco, 2014; Meftahi et al., 2020).

### The complexity of regulation network

Specialized autophagy can manipulate self-antigens and microbial antigens, which mediates antigenic epitope presentation and the functional activity performed by MHC class I/CD8^+^ T cells and MHC class II/CD4^+^ T cells, respectively. The autophagy pathway modulated by diverse factors such as IFNs, NLRs, downstream effectors, and substrates may skew the immune response to induce immunoevasion and immunosuppression. There is a complex network to regulate autophagy, which impacts on the performance of antimicrobial immunity. If we consider microbial invasion as the initial input and antimicrobial immunity as the integrative output, the regulation of autophagy is pivotal to controlling the host–pathogen balance. Up to now, the regulation of the autophagic process is not fully understood. Moreover, autophagy is related to distinct mechanisms such as oxidative stress, energy metabolism, protein synthesis, and apoptosis. All those mechanisms should be comprehensively considered since they are not in vectorial or parallel ways. A long-term effort is needed to clarify the details of autophagy regulation.

### Perspective

The invasion of microbial pathogens activates autophagy in host cells. Autophagy activation modifies downstream immune responses. Due to the duality of autophagy, antimicrobial immunity may be enhanced or weakened. The outcomes of autophagy-mediated immune response are varied, depending on the categories of microbial pathogens and the types of infected cells. Therefore, the antimicrobial application of the autophagy mechanism must consider pathogen characteristics as well as cell types. According to the pathological condition of microbial infection, different schemes of autophagic enhancement/inhibition are carried out. The administration of autophagy modulators (e.g. chloroquine and hydroxychloroquine) has shown therapeutic benefits in certain infectious diseases (e.g. malaria, amebiasis, and Q fever). Furthermore, the mTOR pathway is the potential target against viral pathogens. Yet, mTOR also participates in the replication and release of virions (Maiese, 2020). Autophagy modulators can be combined with immunomodulators based on the mechanistic autophagy–immunity axis (Wang et al., 2018). Hopefully, the best therapeutic effect may be achieved through drug combination.

## Summary

Autophagy is the core mechanism of regulating antimicrobial immunity. Functional analysis shows that autophagy can control pathophysiological manifestations of microbial infection and determine the choice of clinical treatment protocols, especially in the case of coinfection. Different infections or different stages of the same infection require respective protocols, considering the dual nature of autophagy. A few therapeutic agents, based on the autophagy mechanism, have been used in clinical practice. The accumulated evidence confirms their positive effects. There is a bright future for the development of autophagy-related drugs against microbial infections.

## Notes

**Author contributions:** C.Q. and K.W. conceived and designed the manuscript. Y.L., L.B., and K.W. were responsible for data collection and analysis. Additional data were provided by Y.L. and L.B. K.W. wrote the first draft, which was revised by C.Q. All authors approved the manuscript.
